# Patterns of e-cigarette use by sex and age in Thailand: The National Health Examination Survey 2024–2025

**DOI:** 10.18332/tid/218627

**Published:** 2026-07-08

**Authors:** Roengrudee Patanavanich, Wichai Aekplakorn, Suwat Chariyalertsak, Sauwanan Bumrerraj, Polathep Vichitkunakorn, Montakarn Chuemchit, Nareemarn Neelapaichit, Stanton A. Glantz

**Affiliations:** 1 Ramathibodi Hospital, Faculty of Medicine, Mahidol University, Bangkok, Thailand; 2 Faculty of Public Health, Chiang Mai University, Chiang Mai, Thailand; 3 Faculty of Medicine, Khon Kaen University, Khon Kaen, Thailand; 4 Faculty of Medicine, Prince of Songkla University, Hat Yai, Thailand; 5 College of Public Health Sciences, Chulalongkorn University, Bangkok, Thailand; 6 University of California, San Francisco, United States

**Keywords:** gender, Thailand, e-cigarette, NHES

## Abstract

**INTRODUCTION:**

Since their introduction in the early 2000s, the prevalence of e-cigarette use has increased globally. E-cigarette use among females, while usually lower than that of males, is rising and typically higher than cigarette use. This study describes the prevalence of e-cigarette use in Thailand stratified by age and sex, including frequency of use, place obtained, preferred flavors, and reasons for use.

**METHODS:**

This cross-sectional study used the seventh Thai National Health Examination Survey (NHES), conducted between 2024 and 2025. Weighted percentages with 95% confidence intervals (CIs) were used to describe self-reported e-cigarette use patterns across age groups and sex, accounting for survey weights and the complex survey design. We also used multivariable logistic regression models to examine the factors associated with e-cigarette use.

**RESULTS:**

Current e-cigarette use was highest among males aged 20–29 years (13.4%; 95% CI 10.7–16.1). Among current e-cigarette users, dual use was higher among males than females (50.3%; 95% CI: 45.1–55.5 vs 18.8%; 95% CI: 11.7–26.0; p<0.01). Never smoking cigarettes among participants who currently use e-cigarettes was higher among females <30 years than males (64.7%; 95% CI: 55.4–74.1 vs 22.5%; 95% CI: 17.4–27.6; p<0.001). Fruit flavors were the most preferred among e-cigarette users across age groups and sexes. Females were more likely to use e-cigarettes to relieve stress and generally initiated using e-cigarettes at a younger age than males. Multivariable logistic regression revealed that age and sex were significantly (p<0.001) associated with ever, current, and daily e-cigarette use.

**CONCLUSIONS:**

This study describes patterns of e-cigarette use in a country where e-cigarettes are prohibited. The high proportion of dual use and the high prevalence of e-cigarette use among younger adults who never smoked, suggest that e-cigarette use is not primarily motivated by cessation among younger users. Health initiatives should dispel e-cigarette harm reduction claims and denormalize social norms that encourage their use.

## INTRODUCTION

Since the introduction of e-cigarettes in the early 2000s, the prevalence of e-cigarette use has increased globally^1^. Trends in tobacco use among young people have shifted from smoking to vaping, particularly after the introduction of disposable e-cigarettes in 2021^2^. According to the World Health Organization (WHO), several countries have observed greater rates of e-cigarette use among young people than cigarette smoking, with the difference being up to three times^3^. Moreover, due to the attractiveness of product designs and marketing tactics that target youth, the age at initiation of e-cigarette use is decreasing^4^. While most national surveys indicate that e-cigarette use is more common among men, e-cigarette use among women is also on the rise and is usually higher than traditional cigarette use^5^ with a narrowing of the gender gap in tobacco use after e-cigarettes became available^6^. Furthermore, the prevalence of dual use (using both cigarettes and e-cigarettes) is increasing and becoming a common pattern among people who use e-cigarettes^7^.

Epidemiological surveillance and patterns of e-cigarette use are critical for policymakers to design, maintain, and adjust e-cigarette regulation and evaluate policies to prevent e-cigarette use from becoming epidemic. Thailand has completely prohibited e-cigarettes since 2015^8^. In 2025, 42 countries had completely banned e-cigarettes^9^, up from 25 countries in 2016^10^. The most recent national survey on e-cigarette use among Thai adults, conducted in 2021, found that the prevalence of current e-cigarette use among Thai adults aged ≤15 years was 0.14%^11^. However, the Global Youth Tobacco Survey (GYTS) in 2022 reported that the prevalence of current e-cigarette use among Thai students aged 13–15 years was 17.6%, up from 3.3% in 2015^12^.

Most studies that examine the patterns of e-cigarette use were conducted in high-income countries where e-cigarettes were legal^1^. Studies in countries where e-cigarettes are prohibited, especially in low- and middle-income countries (LMICs), are limited and dated^7^. This study used the 7th Thai National Health Examination Survey (NHES VII), conducted between August 2024 and April 2025. The objectives of this study are to analyze the prevalence of cigarette, e-cigarette, and dual use among the Thai population stratified by age and sex, to describe the overall patterns of e-cigarette use in terms of frequency, place obtained, preferred flavors, and reasons for use, and to examine factors associated with e-cigarette use.

## METHODS

### Data source

The NHES is a cross-sectional survey conducted every 5 years among the Thai population aged ≥1 year, using a multistage, stratified sampling technique. In the first stage, five provinces were selected from each of the four regions of Thailand using multistage stratified random sampling, resulting in a total of 20 provinces and Bangkok^13^. In the second stage, 3 to 5 districts were sampled from each province based on population size^13^. The third stage involves selecting villages in rural areas and election units in urban areas. The final stage involved selecting individuals aged ≥1 years, resulting in a total of 32400 participants^13^. The overall response rate was 93%^13^. The total sample used in this study included 25495 participants aged ≥10 years who completed an in-person interview survey on tobacco use. Informed consent was obtained from all participants, and parental consent was obtained for participants aged <18 years. The study was approved by the Ramathibodi Ethical Review Board (COA. MURA2025/701).

### Measures

Ever e-cigarette and combustible cigarette smoking were assessed with the yes/no questions: ‘Have you ever tried e-cigarettes?’ and ‘Have you ever tried smoking?’. Current e-cigarette use and cigarette smoking were assessed with the questions: ‘Have you used e-cigarettes in the last 30 days?’ and ‘Have you smoked cigarettes in the last 30 days?’. Daily e-cigarette use and cigarette smoking were defined as participants who used the products every day in the past 30 days. Dual use was defined as participants who were current users of both combustible cigarettes and e-cigarettes.

E-cigarette preferred flavors were assessed with the question: ‘Which e-cigarette flavors have you used in the past 30 days?’ (mint/menthol, fruit, candy/dessert, and tobacco). Places for obtaining e-cigarettes were assessed with the question: ‘The last time you vaped, where did you get/buy an e-cigarette?’ (flea market/stall, online, vape shop, and friends). Reasons for e-cigarette use were assessed with the question: ‘What were the reasons why you used e-cigarettes?’ (quit smoking, less harmful than cigarettes, reduce stress, friends’ use, enjoy flavors, and attractive/fashionable). Frequency of e-cigarette use was assessed with the question: ‘On average, how many puffs per day do you use e-cigarettes?’.

Other covariates included age, sex, residence (urban, rural), region (north, central, northeast, south, and Bangkok), and household wealth index. A household wealth index variable was developed to indicate socio-economic status. It was categorized as quantiles 1–5, with the lower quintiles indicating less wealth^14^.

### Statistical analysis

We used the NHES-provided weights and stratification variables to account for the multistage sampling design and non-response, matching sample characteristics to national estimates. Descriptive statistics were presented as weighted percentages or proportions for categorical variables with 95% confidence intervals (CIs), and as weighted means with standard deviations or medians with interquartile ranges for continuous variables. The statistics were stratified by age group (10–19, 20–29, and ≥30 years) and sex, to examine variations in e-cigarette use patterns across age and sex. Chi-squared statistics (for categorial variables) and independent t-tests (for continuous variables) were used to compare the difference between two estimates. We also used multivariable logistic regression models to examine the factors associated with e-cigarette use. The statistical analysis was conducted using the svyset command in STATA 17. A p<0.05 was considered statistically significant.

## RESULTS

Of the 25495 participants, 10690 (47.8%) were male, and 14805 (52.2%) were female (Table 1). The median age was 43 (IQR: 27–58) years; 12.7% were aged 10–19 years, 16.1% were aged 20–29 years, and 71.2% were ≥30 years; 46.4% of participants were urban residents, 53.6% were in rural areas, and 19.7% lived in the poorest quintile of the household wealth index. Of all participants, 6223 (28.1%) had ever smoked cigarettes, 2912 (14.7%) currently smoked cigarettes, and 2096 (10.5%) smoked cigarettes daily; 1551 (8.6%) of participants had ever used e-cigarettes, 479 (2.7%) currently used e-cigarettes, and 171 (1.0%) used e-cigarettes daily. Among participants who currently used e-cigarettes, 210 (44.3%) were dual users, and 143 (30.1%) had never smoked cigarettes.

**Table 1:** Characteristics of participants aged ≥10 years, Thailand, 2024–2025 (N=25495)

*Characteristics*	n (%)*
**Sex**	
Male	10690 (47.8)
Female	14805 (52.2)
**Age** (years), median (IQR)*	43 (27–58)
**Age** (years)	
10–19	4341 (12.7)
20–29	2198 (16.1)
≥30	18956 (71.2)
**Residence**	
Urban	13888 (46.4)
Rural	11607 (53.6)
**Region**	
North	5440 (15.9)
Central	5780 (31.9)
Northeast	3882 (25.4)
South	4833 (13.6)
Bangkok	1990 (13.2)
**Wealth index** (quintile)	
1 (lowest)	4925 (19.7)
2	5086 (19.7)
3	5078 (20.4)
4	4984 (19.8)
5 (highest)	4885 (20.4)
**Cigarette use**	
Ever	6223 (28.1)
Current	2912 (14.7)
Daily	2096 (10.5)
**E-cigarette use**	
Ever	1551 (8.6)
Current	479 (2.7)
Daily	171 (1.0)
**Dual use among current e-cigarette users**	210 (44.3)
**Never smoking among current e-cigarette users**	143 (30.1)
**Age at initiation of e-cigarette use** (years)	
<15	338 (12.7)
15–19	406 (28.6)
20–29	413 (33.9)
≥30	356 (24.8)

* Data presented in weighted percentage (%) and weighted median and interquartile range (IQR).

### Patterns of cigarette and e-cigarette use by age

The prevalence of current combustible cigarette smoking among the Thai population aged ≥10 years was 14.7% (95% CI: 13.7–15.7), and current e-cigarette use was 2.7% (95% CI: 2.4–3.1) (population weighted results; Table 2). The prevalence of current combustible cigarette smoking was higher than current e-cigarette use in all age groups except adolescents (10–19 years), where it was about the same. The prevalence of ever e-cigarette use among adolescents (10–19 years) was significantly higher than ever combustible cigarette smoking (14.8%: 95% CI: 13.4–16.1 vs 11.5%: 95% CI: 10.5–12.5, p<0.01), but not among other age groups. The prevalence of current e-cigarette use was highest among those aged 20–29 years (8.3%: 95% CI: 6.9–9.7), then among those aged 10–19 years (5.3%: 95% CI: 4.4–6.1), and lowest among those aged ≥30 years (1.0%: 95% CI: 0.7–1.4). The proportion of participants who reported current use of both e-cigarettes and combustible cigarettes (dual use) was 44.3% (95% CI: 39.8–48.7) and increased as the age increased, from 38.1% (95% CI: 31.1–45.1) among those aged 10–19 years to 50.0% (95% CI: 40.9–59.1) among those aged ≥30 years. The proportion of participants who currently used e-cigarettes but never smoked cigarettes was 30.1% (95% CI: 26.0–34.2) and increased as the age decreased, from 23.9% (95% CI: 16.2–31.7) among those aged ≥30 years to 37.1% (95% CI: 30.1–44.0) among those aged 10–19 years.

**Table 2:** Patterns of e-cigarette use by age of the participants, Thailand, 2024–2025 (N=25495)*

*Patterns of use*	*All % (95% CI)*	*10–19 years % (95% CI)*	*20–29 years % (95% CI)*	*≥30 years % (95% CI)*
**Cigarette use**				
Ever	28.1 (26.7–29.6)	11.5 (10.5–12.5)	31.3 (28.7–33.8)	30.4 (28.7–32.0)
Current	14.7 (13.7–15.7)	5.4 (4.7–6.2)	19.4 (17.4–21.4)	15.3 (14.1–16.4)
Daily	10.5 (9.7–11.3)	2.4 (1.8–3.0)	13.2 (11.7–14.6)	11.3 (10.3–12.3)
**Sole cigarette use**	13.5 (12.4–14.5)	3.4 (2.7–4.1)	15.7 (13.8–17.6)	14.7 (13.6–15.9)
**Dual use among current cigarette users**	8.3 (7.3–9.3)	37.0 (29.7–44.2)	18.9 (14.9–22.8)	3.4 (2.7–4.2)
**E-cigarette use**				
Ever	8.6 (7.9–9.3)	14.8 (13.4–16.1)	22.9 (20.8–25.0)	4.3 (3.5–5.1)
Current	2.7 (2.4–3.1)	5.3 (4.4–6.1)	8.3 (6.9–9.7)	1.0 (0.7–1.4)
Daily	1.0 (0.9–1.2)	1.7 (1.3–2.1)	3.3 (2.6–4.0)	0.4 (0.3–0.6)
**Sole e-cigarette use**	1.5 (1.3–1.7)	3.3 (2.6–4.0)	4.6 (3.7–5.5)	0.5 (0.4–0.7)
**Dual use among current e-cigarette users**	44.3 (39.8–48.7)	38.1 (31.1–45.1)	44.2 (36.8–51.6)	50.0 (40.9–59.1)
**Never smoking among current e-cigarette users**	30.1 (26.0–34.2)	37.1 (30.1–44.0)	30.1 (23.3–36.9)	23.9 (16.2–31.7)
**Dual use**	1.2 (1.1–1.3)	2.0 (1.6–2.4)	3.7 (2.9–4.4)	0.5 (0.4–0.6)
**Age at initiation of e-cigarettes** (years), mean (95% CI)	23.7 (23.2–24.2)	14.3 (14.2–14.5)	20.2 (19.9–20.5)	33.9 (33.1–34.7)
**Frequency of use** (times/day), mean (95% CI)	29.9 (19.9–39.9)	19.1 (13.0–25.2)	42.8 (19.6–65.9)	16.9 (12.8–20.9)
**Flavor preferred**				
Mint/menthol	30.0 (25.9–34.2)	33.3 (26.6–40.1)	27.7 (21.0–34.4)	31.3 (22.8–39.8)
Fruit	73.3 (69.3–77.2)	67.8 (61.1–74.5)	75.4 (69.0–81.8)	74.3 (66.3–82.3)
Candy/dessert	9.5 (6.9–12.1)	17.7 (12.2–23.2)	8.5 (4.3–12.6)	4.0 (0.4–7.5)
Tobacco/other	2.2 (0.8–3.5)	2.7 (0.4–5.0)	1.9 (0.0–3.9)	2.2 (0.0–4.8)
**Place for obtaining e-cigarettes**				
Flea market/stall	13.9 (10.8–17.0)	16.4 (11.1–21.8)	13.3 (8.2–18.3)	12.7 (6.6–18.8)
Online	41.0 (36.6–45.4)	31.3 (24.7–38.0)	46.4 (39.0–53.9)	39.9 (31.0–48.9)
Vape shop (illegal)	20.1 (16.5–23.7)	17.2 (11.8–22.7)	20.4 (14.4–26.4)	22.0 (14.4–29.5)
Friends	19.6 (16.0–23.2)	26.9 (20.5–33.3)	15.3 (10.0–20.7)	20.6 (13.3–28.0)
**Reason for use**				
Quit smoking	15.1 (11.9–18.3)	5.9 (2.5–9.3)	16.9 (11.3–22.5)	20.2 (12.8–27.5)
Less harmful than cigarettes	6.9 (4.6–9.2)	4.5 (1.5–7.5)	7.3 (3.4–11.2)	8.4 (3.3–13.5)
Reduce stress	19.1 (15.6–22.7)	25.2 (18.9–31.4)	19.0 (13.2–24.9)	13.9 (7.6–20.3)
Friends’ use	52.7 (48.2–57.2)	71.2 (64.7–77.8)	48.9 (41.5–56.4)	42.8 (33.7–51.8)
Enjoy flavors	42.8 (38.3–47.2)	35.7 (28.8–42.6)	42.3 (34.9–49.7)	50.0 (40.8–59.1)
Attractive/fashionable	11.7 (8.8–14.6)	19.0 (13.3–24.6)	11.4 (6.6–16.1)	5.9 (1.6–10.2)

* Data presented in weighted percentage (%).

The most commonly preferred flavors were fruits (73.3%) and mint/menthol (30.0%), with tobacco flavor the least preferred (2.2%).

Among participants who currently use e-cigarettes, the commonly stated reasons for e-cigarette use were friends’ use (52.7%), followed by enjoyment of flavors (42.8%), and reducing stress (19.1%). However, among individuals aged 10–19 years, the most common reason for e-cigarette use was friends’ use (71.2%). Using e-cigarettes based on friends’ recommendations and reducing stress were most common among those aged 10–19 years and lowest among those aged ≥30 years. In contrast, quitting smoking as the reason for use was highest among those aged ≥30 years and lowest among those aged 10–19 years (Table 2). E-cigarettes were obtained mostly from online (41.0%). The second most common place to obtain e-cigarettes was illegal vape shops among those aged ≥20 years, but from friends among those aged 10–19 years.

### Patterns of cigarette and e-cigarette use by age and sex

The overall prevalence of current combustible cigarette smoking was 28.9% (95% CI: 27.1–30.7) among males and 1.6% (95% CI: 1.3–1.9) among females (population weighted results; Table 3). Prevalence of current e-cigarette use was 4.6% (95% CI: 3.9–5.3) among males and 1.0% (95% CI: 0.8–1.2) among females. Also, the prevalence of current e-cigarette use was highest among males 20–29 years old (13.4%; 95% CI: 10.7–16.1) and females 10–19 years old (3.4%; 95% CI: 2.5–4.4) (Table 3). The prevalence of current e-cigarette use was lowest among those aged ≥30 years in both sexes. Prevalence of current e-cigarettes among younger females (aged 10–19 years) was higher than combustible cigarettes (3.4%; 95% CI: 2.5–4.4 vs 1.0%; 95% CI: 0.6–1.4, p<0.001). Dual use among current e-cigarette users was more prevalent among males than females (50.3%; 95% CI: 45.1–55.5 vs 18.8%; 95% CI: 11.7–26.0, p<0.001).

**Table 3:** Patterns of e-cigarette use by age and sex, Thailand, 2024–2025 (N=25495)*

*Patterns of use*	*All % (95% CI)*		*10–19 years % (95% CI)*		*20–29 years % (95% CI)*		*≥30 years % (95% CI)*	
	*Male*	*Female*	*Male*	*Female*	*Male*	*Female*	*Male*	*Female*
**Cigarette use**								
Ever	51.9 (49.8–54.1)	6.3 (5.1–7.4)	19.3 (17.6–21.0)	3.5 (2.7–4.2)	54.1 (49.9–58.2)	8.5 (6.2–10.8)	57.7 (55.3–60.2)	6.3 (5.2–7.4)
Current	28.9 (27.1–30.7)	1.6 (1.3–1.9)	9.7 (8.4–11.0)	1.0 (0.6–1.4)	37.0 (33.4–40.7)	1.7 (1.0–2.4)	30.7 (28.6–32.7)	1.7 (1.3–2.1)
Daily	21.0 (19.6–22.4)	0.8 (0.7–1.0)	4.6 (3.5–5.7)	0.1 (-0.1–0.3)	25.8 (23.0–28.6)	0.6 (0.2–0.9)	23.1 (21.2–24.9)	1.0 (0.8–1.2)
**Sole cigarette use**	26.6 (24.6–28.5)	1.4 (1.1–1.7)	6.2 (5.1–7.4)	0.5 (0.2–0.8)	30.4 (26.8–34.0)	1.0 (0.6–1.5)	29.6 (27.4–31.8)	1.7 (1.3–2.1)
**Dual use among current cigarette users**	8.1 (7.0–9.1)	11.8 (7.5–16.0)	35.6 (28.0–43.2)	50.3 (26.5–74.1)	17.9 (14.0–21.9)	38.7 (17.3–60.0)	3.5 (2.7–4.3)	2.1 (0.0–4.2)
**E-cigarette use**								
Ever	14.3 (13.2–15.4)	3.4 (3.0–3.8)	19.3 (17.5–21.0)	10.1 (8.5–11.7)	36.7 (33.0–40.3)	9.1 (7.4–10.9)	8.0 (6.6–9.3)	1.1 (0.8–1.4)
Current	4.6 (3.9–5.3)	1.0 (0.8–1.2)	7.0 (5.7–8.3)	3.4 (2.5–4.4)	13.4 (10.7–16.1)	3.2 (2.3–4.0)	2.1 (1.4–2.7)	0.2 (0.1–0.3)
Daily	1.8 (1.5–2.1)	0.4 (0.3–0.5)	2.3 (1.6–3.0)	1.1 (0.7–1.5)	5.3 (3.9–6.7)	1.2 (0.7–1.7)	0.8 (0.6–1.1)	0.1 (0.0–0.1)
**Sole e-cigarette use**	2.3 (1.9–2.7)	0.8 (0.6–1.0)	3.6 (2.8–4.4)	2.9 (1.9–3.9)	6.7 (5.1–8.4)	2.5 (1.7–3.3)	1.0 (0.7–1.3)	0.1 (0.0–0.2)
**Dual use among current e-cigarette users**	50.3 (45.1–55.5)	18.8 (11.7–26.0)	49.0 (40.0–57.9)	15.1 (6.4–23.9)	49.6 (41.3–58.0)	20.9 (7.4–34.4)	52.5 (42.5–62.0)	23.7 (1.4–26.1)
**Never smoking among current e-cigarette users**	23.0 (18.6–27.3)	60.1 (51.2–69.0)	23.5 (15.9–31.1)	65.6 (54.0–77.2)	22.1 (15.2–29.0)	64.0 (48.1–79.9)	24.1 (15.7–32.4)	22.7 (0.7–44.7)
**Dual use**	2.3 (2.0–2.6)	0.2 (0.1–0.3)	3.4 (2.7–4.2)	0.5 (0.2–0.8)	6.6 (5.1–8.2)	0.7 (0.2–1.1)	1.1 (0.8–1.3)	0.0 (0.0–0.1)
**Age at initiation of e-cigarettes** (years), mean (95% CI)	24.4 (23.9–25.0)	20.9 (20.1–21.7)	14.4 (14.2–14.6)	14.2 (14.0–14.5)	20.2 (19.8–20.5)	20.5 (20.0–21.0)	34.1 (33.5–34.8)	32.4 (30.5–34.1)
**Frequency of use** (times/day), mean (95% CI)	33.6 (21.0–46.3)	14.2 (10.0–18.5)	20.0 (11.5–28.5)	17.3 (10.0–24.6)	49.9 (21.1–78.7)	12.3 (6.2–18.4)	17.3 (12.9–21.8)	11.4 (4.6–18.1)
**Flavor preferred**								
Mint/menthol	29.4 (24.8–34.1)	30.4 (22.3–38.5)	32.1 (23.7–40.4)	36.0 (24.3–47.7)	26.6 (19.2–33.9)	32.5 (17.0–48.1)	32.1 (23.4–40.9)	9.7 (-2.7–22.1)
Fruit	73.4 (68.9–77.9)	65.7 (57.3–74.0)	64.7 (56.1–73.2)	74.4 (63.7–85.0)	79.2 (72.4–86.0)	59.4 (43.0–75.7)	70.1 (61.5–78.7)	62.5 (42.1–82.7)
Candy/dessert	9.2 (6.2–12.1)	9.7 (4.5–14.9)	17.6 (10.8–24.4)	17.9 (8.5–27.2)	9.5 (4.6–14.4)	3.9 (-2.5–10.3)	3.3 (-0.1–6.7)	6.5 (-0.4–16.9)
Tobacco/other	2.5 (0.9–4.1)	1.5 (-0.7–3.7)	4.0 (0.5–7.5)	n/a	1.6 (-0.5–3.7)	3.1 (-2.7–8.9)	3.0 (-0.3–6.3)	n/a
**Place for obtaining e-cigarettes**								
Flea market/stall	13.9 (10.4–17.5)	13.6 (7.4–20.0)	16.2 (9.6–22.8)	17.0 (7.8–26.1)	13.4 (7.7–19.1)	12.7 (1.6–23.7)	13.3 (6.7–20.0)	5.4 (-6.5–17.3)
Online	41.7 (36.6–46.8)	38.1 (29.3–47.0)	34.3 (25.8–42.8)	25.1 (14.5–35.6)	46.5 (38.2–54.8)	46.2 (29.7–62.8)	39.0 (29.4–48.5)	51.2 (25.0–77.5)
Vape shop (illegal)	20.5 (16.3–24.6)	18.3 (11.3–25.4)	18.7 (11.7–25.7)	14.1 (5.6–22.6)	19.5 (12.9–26.1)	24.3 (10.0–38.5)	23.1 (14.9–31.4)	7.9 (-6.3–22.0)
Friends	18.7 (14.7–22.8)	23.3 (15.5–31.0)	24.6 (16.8–32.3)	31.9 (20.6–43.3)	15.7 (9.7–21.8)	13.6 (2.2–25.0)	19.6 (11.8–27.3)	33.4 (8.6–58.2)
**Reason for use**								
Quit smoking	16.0 (12.2–19.8)	11.4 (5.6–17.2)	5.1 (1.2–9.1)	7.6 (1.1–14.1)	18.5 (12.0–25.0)	9.9 (0.0–19.9)	19.2 (11.5–26.9)	31.5 (7.1–55.9)
Less harmful than cigarettes	7.2 (4.5–9.9)	5.7 (1.5–9.9)	2.4 (-0.3–5.2)	8.8 (1.9–15.6)	8.1 (3.5–12.6)	4.0 (-2.5–10.5)	9.0 (3.4–14.6)	1.6 (-5.0–8.2)
Reduce stress	15.2 (11.4–18.9)	35.9 (27.1–44.6)	18.4 (11.5–25.4)	39.3 (27.4–51.2)	15.0 (9.0–21.0)	36.1 (20.1–52.0)	13.2 (6.6–20.0)	22.3 (0.4–44.1)
Friends’ use	51.0 (45.9–56.2)	59.7 (50.8–68.7)	69.1 (60.8–77.4)	75.7 (65.3–86.2)	49.5 (41.2–57.9)	46.3 (29.8–62.9)	41.4 (31.8–51.0)	59.1 (33.2–84.9)
Enjoy flavors	43.9 (38.8–49.1)	37.8 (29.0–46.6)	36.1 (27.5–44.7)	34.8 (23.1–46.4)	41.8 (33.6–50.1)	44.4 (27.9–60.9)	52.5 (42.7–62.2)	20.3 (-0.8–41.4)
Attractive/fashionable	11.2 (7.9–14.4)	14.1 (7.7–20.4)	20.2 (13.0–27.4)	16.4 (7.3–25.4)	10.4 (5.3–15.5)	15.4 (3.4–27.3)	6.4 (1.6–11.2)	n/a

* Data presented in weighted percentage (%), n/a: not available.

The proportions of dual use among current e-cigarette use in females increased with age, from 15.1% (95% CI: 6.4–23.9) among those aged 10–19 years to 23.7% (95% CI: 1.4–26.1) among those ≥30 years. The proportion of current e-cigarette use who had never smoked was more prevalent among females younger than 30 years old than males (64.7%; 95% CI: 55.4–74.1 vs 22.5%; 95% CI: 17.4–27.6, p<0.001) (results for combined 10–19 and 20–29 age groups in Table 2). In terms of frequency of use, males reported a mean of 33.6 puffs/day (95% CI: 21.0–46.3), compared to 14.2 puffs/day (95% CI: 10.0–18.5) in females.

Fruit flavors were the most preferred e-cigarette flavors across gender and age groups. Females are likely to use e-cigarettes to relieve stress than males (35.9%; 95% CI: 27.1–44.6 vs 15.2%; 95% CI: 11.4–18.9, p<0.001) and generally start using e-cigarettes at a younger age (20.9 years; 95% CI: 20.1–21.7 vs 24.4 years; 95% CI: 23.9–25.0, p<0.001). Unlike other sexes and age groups, where the most common source of e-cigarettes was online platforms, young females (aged 10–19 years) most likely obtained their e-cigarettes from friends (31.9%).

### Factors associated with e-cigarette use

The multivariable logistic regression (Table 4) revealed that men were more likely than women to use e-cigarette (ever: OR=5.04; 95% CI: 4.26–5.96; p<0.001, current: OR=4.69; 95% CI: 3.57–6.17; p<0.001, daily: OR=4.80; 95% CI: 3.03–7.58; p<0.001, and dual use: OR=4.03; 95% CI: 2.02–8.04; p<0.001). However, men who had never smoked cigarettes were less likely than women to become current e-cigarette users (OR=0.22; 95% CI: 0.12–0.41; p<0.001). E-cigarette use (ever: OR=0.94; 95% CI: 0.94–0.95; p<0.001, current: OR=0.94; 95% CI: 0.93–0.94; p<0.001, and daily use: OR=0.94; 95% CI: 0.94–0.95; p<0.001) decreased with increasing age. Urbanicity and wealth were generally not associated with e-cigarette use. Current e-cigarette use was the highest in Bangkok.

**Table 4:** Factors associated with e-cigarette use, Thailand, 2024–2025 (N=25495)

*Variables*	*Ever e-cigarette use*			*Current e-cigarette use*			*Daily e-cigarette use*			*Dual use among current e-cigarette users*			*Never smoking among current e-cigarette users*		
	*AOR*	*95% CI*	*p*	*AOR*	*95% CI*	*p*	*AOR*	*95% CI*	*p*	*AOR*	*95% CI*	*p*	*AOR*	*95% CI*	*p*
**Sex**															
Male	5.04	4.26–5.96	<0.001	4.69	3.57–6.17	<0.001	4.80	3.03–7.58	<0.001	4.03	2.02–8.04	<0.001	0.22	0.12–0.41	<0.001
Female (ref.)															
**Age** (years)	0.94	0.94–0.95	<0.001	0.94	0.93–0.94	<0.001	0.94	0.94–0.95	<0.001	1.01	0.99–1.03	0.355	0.99	0.96–1.02	0.456
**Residence**															
Urban	1.12	0.95–1.32	0.191	1.31	1.00–1.72	0.052	0.96	0.64–1.45	0.846	0.99	0.57–1.70	0.96	0.79	0.44–1.42	0.426
Rural (ref.)															
**Region**															
North	0.92	0.69–1.22	0.568	0.47	0.30–0.75	0.002	0.55	0.25–1.20	0.132	0.94	0.40–2.24	0.894	0.42	0.14–1.33	0.143
Central	0.77	0.59–1.01	0.056	0.65	0.44–0.97	0.035	0.91	0.50–1.68	0.77	0.72	0.32–1.60	0.418	1.27	0.47–3.42	0.631
Northeast	0.77	0.58–1.03	0.076	0.62	0.40–0.96	0.031	0.55	0.26–1.15	0.113	0.63	0.28–1.40	0.259	1.19	0.48–2.98	0.71
South	0.3	0.22–0.42	<0.001	0.33	0.20–0.56	<0.001	0.44	0.20–0.99	0.046	1.11	0.44–2.78	0.829	1.08	0.36–3.23	0.884
Bangkok (ref.)															
**Wealth index** (quintiles)															
1 (lowest)	1.10	0.85–1.42	0.465	1.30	0.86–1.96	0.215	0.61	0.30–1.25	0.176	1.42	0.64–3.16	0.389	1.34	0.54–3.34	0.528
2	1.08	0.83–1.40	0.576	1.25	0.81–1.95	0.315	1.07	0.56–2.02	0.847	1.55	0.66–3.63	0.311	0.85	0.32–2.29	0.748
3	0.96	0.75–1.24	0.779	1.17	0.79–1.74	0.434	1.04	0.60–1.81	0.899	1.03	0.46–2.32	0.934	1.12	0.46–2.72	0.797
4	1.39	1.09–1.77	0.007	1.09	0.74–1.60	0.671	1.16	0.68–1.98	0.578	1.34	0.62–2.89	0.461	1.03	0.40–2.67	0.944
5 (highest) (ref.)															

AOR: adjusted odds ratio. Adjusted for all variables shown in the Table.

## DISCUSSION

E-cigarette use is more common among younger Thais than cigarettes, especially among females. The high prevalence of e-cigarette use among young participants who never smoked is also concerning as several studies found that young people who never smoked are initiating nicotine addiction with e-cigarettes^4^. In addition, youth who initiate nicotine use with e-cigarettes are more likely to go on to smoking cigarettes^15^, including Thai adolescents^16^. Over time, this ‘gateway effect’ is associated with increased adolescent and young adult smoking in England and elsewhere^17^.

Only 15.1% (95% CI: 11.9–18.3) of people (and only 5.9% [95% CI: 2.5–9.3] of those aged 10–19 years), used e-cigarettes to stop smoking (Table 2); they started using them when friends suggested them, liked their flavors, and used them to relieve stress, particularly among young females. This finding is consistent with the International Pediatric Association’s conclusion that the rising prevalence of e-cigarette use among young people was driven by deceptive advertising, accessibility, attractive flavors, and product designs^18^.

This finding also contradicts the claims made by the tobacco industry and e-cigarette advocates that e-cigarettes are essential as a cessation strategy for people who currently smoke; this finding is consistent with several studies showing that young people use e-cigarettes out of curiosity rather than to quit smoking^19^. In addition, in the US, youths who use e-cigarettes specifically to quit smoking were associated with significantly lower odds of having stopped smoking cigarettes^20^. Moreover, e-cigarettes as consumer products are not associated with stopping smoking among youth or adults^18,21^. Instead, people who use e-cigarettes are likely to become dual users, which has greater health risks than cigarette smoking alone^18^.

This study also found that the prevalence of cigarette smoking among people who currently used e-cigarettes aged ≥18 years in Thailand (45.3%) was higher than that of other countries, such as the US (39.6%; aged ≥18 years surveyed in 2021–2023)^22^, England (34.2%; aged ≥18 years surveyed in 2024)^23^ and Mexico (31.5%; aged ≥18 years surveyed in 2018–2019)^24^ Unlike the US, England, and Mexico, the likelihood of dual use among the Thai population increased with age^23-25^ (these comparisons should be interpreted with caution because of differences in the time the data were collected and the precise status of the e-cigarette markets). This study found that the 42.3% (95% CI: 36.2–48.5) prevalence of dual use among Thai adolescents aged 15–24 years was higher than the previous survey in 2019 among the same age group (21.3%; 95% CI: 19.3–23.4)^26^. The increasing prevalence of dual use is alarming since dual use is associated with a higher disease risk than just using e-cigarettes or smoking cigarettes^27^.

Although Thailand banned the sale and import of e-cigarettes across all platforms in 2015^8^, this study found that accessing e-cigarettes from online platforms is the most common source across all age groups and sexes. This finding is similar to other countries where e-cigarettes are banned including Mexico^28^, Brazil^29^, and India^30^. Even in countries where e-cigarettes are legal, it is challenging to enforce restrictions on internet retailers to restrict underage access^31^. One major issue is that digital content is cross-border; therefore, service providers may be located outside of the country where the service is accessed. Although international cooperation is critical to successfully controlling cross-border digital marketing, existing national regulations must be strengthened and maintained, such as strictly making e-cigarettes non-shippable through all postal and other delivery services and closely monitoring the violation of online sale and advertising of e-cigarettes.

In addition, political commitment, stringent implementation, stiff penalties, strong collaboration from relevant agencies of the government, and public awareness are essential to effectively restricting online access to e-cigarettes, particularly among youth. For example, in February 2025, the Thai Prime Minister ordered a serious action against e-cigarette products by targeting major suppliers after three schoolgirls were admitted to the hospital with severe lung complications caused by e-cigarette use^32^. Following the Prime Minister’s order, the authorities claimed that in the first two weeks of the order, the number of e-cigarettes seized and illegal online stores blocked, was substantially greater than the total for the entire previous year, and two months later, the officials claimed that the number of sales and users substantially dropped^33^. Further research to evaluate the effects of this major crackdown on the prevalence of e-cigarette use, especially among youths, is needed.

Friends are another frequent source of e-cigarettes for young people. A study in Australia found that peer influence on e-cigarette use extends beyond peer pressure and includes ‘money-making opportunities’ and ‘social norms’^34^. This situation is also observed in Thailand, where students sold e-cigarettes due to household economic problems, lack of knowledge about the dangers of e-cigarettes, and social structure that led to a lack of family relationships^35^. Teaching refusal skills alone might not be enough to address this emerging social issue that goes beyond peer pressure^34^. Following success with cigarettes, potential strategies are denormalization to make e-cigarettes less socially acceptable by promoting the effects of secondhand exposure to reinforce non-using norms that have had the side effect of reducing smoking^36^. After all, 94.7% of youth and 97.3% of all Thais do not currently use e-cigarettes (Table 2).

### Strengths and limitations

This study has several strengths. It uses Thailand’s most recent nationally representative survey, which includes the population aged ≥10 years. This survey contains a comprehensive set of questionnaires on e-cigarette use, allowing us to explore patterns of e-cigarette use in several aspects. Lastly, this study describes patterns of e-cigarette use in a country where e-cigarettes are banned and can help policymakers to redesign better policies to address e-cigarettes’ problems.

This study has some limitations. Because e-cigarettes are illegal in Thailand, it is possible that some respondents intended to answer in a way that would be socially acceptable, which could lead to underestimates of self-reported e-cigarette use as well as response and recall bias.

## CONCLUSIONS

Patterns of e-cigarette use differ across age and sex. The high proportion of dual use and the high prevalence of e-cigarette use among people who never smoke, among people aged 10–19 years, suggest that e-cigarette use is not primarily motivated by cessation. Instead, social norms and curiosity are the most common reasons to initiate e-cigarette use. It is crucial for health initiatives, including those led by youth, to dispel prevalent misconceptions about the harm reduction claims of e-cigarettes and deformalize social norms that encourage their use.

## Data Availability

The data supporting this research are available from the authors on reasonable request.
